# Water regime-dependent effects of organic amendments on Cd/As accumulation and potential grain risk in rice

**DOI:** 10.3389/fpls.2026.1942050

**Published:** 2026-09-17

**Authors:** Ping Zhang, Bo Li, Jie Zhang, Taowen Pan, Jibiao Geng, Bingbing Song, Jianrong Zhao, Hongbao Wu

**Affiliations:** 1 College of Resources and Environment, Anhui Science and Technology University, Chuzhou, China; 2 College of Resources and Environment, Linyi University, Key Laboratory of Green Functional Fertilizer Innovation, Ministry of Agriculture and Rural Affairs, Linyi, China; 3 Hunan Provincial Ecological Environment Monitoring Center, State Environmental Protection Key Laboratory of Monitoring for Heavy Metal Pollutants, Changsha, China

**Keywords:** arsenic, cadmium, organic amendments, paddy soil, water regime

## Abstract

**Introduction:**

Cadmium (Cd) and arsenic (As) co-contamination in paddy soils poses a persistent challenge for rice safety due to their distinct responses to soil redox conditions. Although organic amendments have been widely used to regulate metal(loid) availability, their effects on Cd/As behavior in soil-rice systems under contrasting water regimes remain unclear.

**Methods:**

A pot experiment was conducted under flooding-moisture alternation (FM) and continuous flooding (F) to evaluate the effects of fulvic acid (FA), humic acid (HA), and woody peat (PT) on Cd/As availability, Cd/As in iron plaque (IP), and grain Cd/As accumulation.

**Results:**

HA reduced soil available Cd under both water regimes, whereas FA decreased it mainly under F. Available As changed little under FM but increased under F, particularly with FA and PT. Water regime also altered Fe_IP_, Cd_IP_, and As_IP_ relationships. Under FM, As_IP_ was positively associated with Fe_IP_, whereas Cd_IP_ showed no significant relationship with Fe_IP_. Under F, both Cd_IP_ and As_IP_ were positively associated with Fe_IP_. The changes in soil availability and IP-associated retention did not directly translate into grain Cd/As accumulation. PT produced the largest simultaneous decreases in grain Cd and As concentrations under FM, by 47.20% and 23.96%, respectively, and resulted in the lowest screening-level grain risk index (GRI). Under F, all amendments reduced grain Cd, with HA showing the greatest effect, whereas only FA also reduced grain As. The GRI decreased across all amendment treatments under F. Grain Cd under FM and grain As under F were mainly linked to root uptake and shoot-to-grain transfer, whereas grain Cd under F and grain As under FM were more related to soil mobility and IP-mediated retention.

**Discussion:**

These findings demonstrate that amendment effects on grain Cd/As concentrations were water-regime dependent: PT simultaneously reduced both elements under FM, whereas under F, HA produced the greatest grain Cd reduction and FA was the only amendment that also reduced grain As. These concentration-based responses require further field validation together with biomass and yield assessment.

## Graphical Abstract

**Figure:**
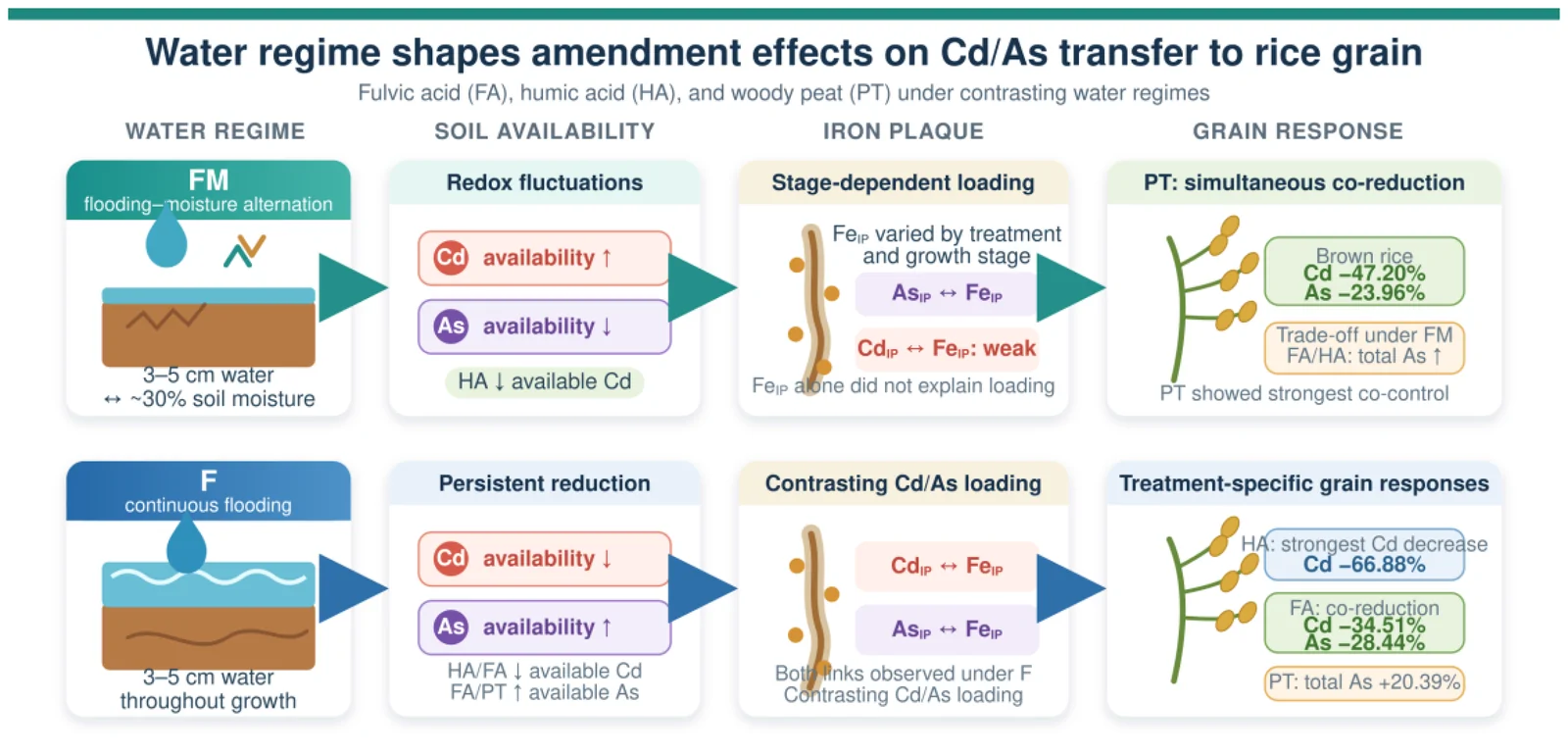

## 1 Introduction

Contamination of paddy soils with cadmium (Cd) and arsenic (As), two toxic metal(loid)s, has become a persistent concern for rice safety and human health (Zou et al., 2021; Hou et al., 2025). In paddy soils, the co-occurrence of Cd/As is commonly associated with geological sources, including metal-rich parent materials and mineral weathering, as well as anthropogenic inputs such as mining, smelting, and industrial emissions (Li et al., 2023; Guo et al., 2026). Rice (*Oryza sativa* L.) is a major dietary source of Cd/As exposure in many rice-consuming regions, owing to its capacity to take up Cd/As from soils and accumulate them in grains (Sen et al., 2020; Zhao and Wang, 2020). Nevertheless, simultaneous regulation of Cd/As in paddy soils remains difficult because of their distinct responses to redox fluctuations (Mabagala et al., 2024). Such opposite changes in soil Cd/As create a major challenge for developing a single management strategy that could concurrently reduce Cd and As uptake by rice.

Water management has been widely adopted to control Cd/As mobility and transfer in the soil–rice system (Honma et al., 2016; Hao et al., 2026). Paddy fields are commonly managed under continuous flooding or flooding–moisture alternation, both of which could markedly affect soil pH, redox potential (Eh), Fe/Mn cycling, and Cd/As mobility (Wang et al., 2022). Previous studies showed that continuous flooding may decrease soil Cd availability and subsequent Cd accumulation in rice, whereas it may promote the release of soil-bound As into soil solution and increase As mobility (Qiao et al., 2019; Li et al., 2026). Intermittent flooding or flooding–moisture alternation can limit As mobilization through periodic re-oxidation, whereas the redox fluctuations may also increase Cd availability through sulfide oxidation and the transformation of Fe/Mn oxides (Honma et al., 2016; Wang et al., 2022). Such redox-driven trade-offs indicate that reliable Cd/As co-control may require the integration of water regime regulation with suitable soil amendments.

Organic amendments provide a complementary approach for regulating Cd/As behavior in soils by modifying Cd/As availability through pH regulation, dissolved organic matter release, and functional-group complexation (Li et al., 2021; Liu et al., 2024). Humic substances, major components of soil organic matter, may regulate Cd/As mobility through direct metal(loid) complexation and indirect changes in soil physicochemical and biochemical properties (Bai et al., 2018; Jin et al., 2023). Among humic substances, differences in solubility, molecular structure, humification degree, and decomposition behavior between humic acid (HA) and fulvic acid (FA) may lead to distinct effects on Cd/As availability and subsequent transfer in soil–rice systems (Li et al., 2021, 2026). In flooded paddy soils, organic amendments may also function as carbon sources or electron shuttles, stimulating microbial Fe/Mn reduction and the transformation of Fe/Mn oxides, which are important carriers for Cd/As (Xu et al., 2017; Chen et al., 2025). These coupled processes may immobilize Cd by increasing soil pH, enhancing organic binding, or altering Cd into less labile fractions, but may also mobilize As by promoting Fe oxide reduction, ligand competition, and possibly facilitating the reduction of As (V) to the more mobile As (III) (Qiao et al., 2019; Li et al., 2021). Therefore, the humic substances may exert contrasting effects on Cd/As availability in paddy soils, depending strongly on their properties and water regime.

Iron plaque (IP), mainly composed of Fe/Mn (hydr) oxides, commonly forms on rice roots through the oxidation and deposition of Fe(II) in the rhizosphere (Zandi et al., 2023; Yuan et al., 2026). Owing to its high reactivity and large surface area, IP serves as an important soil–root interface that can regulate the transfer of Cd/As from soils to rice roots (Yu et al., 2024; Long et al., 2026). However, the role of IP in Cd/As transfer remains complex under different water regimes because both IP formation and its capacity to retain Cd/As are sensitive to soil redox conditions, Fe/Mn availability, root oxygen release, and rhizosphere chemistry under different water regimes (Zhang et al., 2023; Wu et al., 2025). The organic amendments may further affect IP formation by modifying Fe/Mn reduction, Fe migration, and rhizosphere microenvironments (Liu et al., 2021). In addition, the Cd/As may exhibit distinct apparent affinities, loading patterns, and exchangeability on IP, which can vary with water regime and organic amendments and thereby contribute to the complexity of IP-mediated Cd/As retention (Liu et al., 2021; Wu et al., 2025). However, the joint effects of water regime and organic amendment on IP formation and Cd/As loading on IP remain unclear.

Previous studies have mainly focused on soil available Cd/As, as they represent the labile pools that are more directly related to Cd/As mobility, root accessibility, and potential grain accumulation risk than total Cd/As (Honma et al., 2016; Tan et al., 2023). However, grain Cd/As accumulation is controlled by a series of connected processes, including soil Cd/As availability, IP-mediated Cd/As retention, root uptake, and internal translocation to grains (Jing et al., 2023; Kong et al., 2024). These processes may respond differently to water regime and organic amendments. Therefore, focusing only on soil availability may overlook key processes that determine final grain accumulation, particularly when changes in available Cd/As are limited or decoupled from subsequent IP retention and translocation within rice tissues.

In this study, a pot experiment was conducted under two water regimes to evaluate the effects of three organic amendments on soil Cd/As behavior in the soil–rice system and the associated risk of grain Cd/As accumulation. The objectives were to (i) examine the responses of soil Cd/As availability to organic amendments under different water regimes; (ii) determine the effects of organic amendments on IP formation and IP-mediated Cd/As retention; (iii) clarify the contribution of soil Cd/As availability and IP-mediated Cd/As retention to grain Cd/As accumulation; and (iv) evaluate grain Cd/As risk and identify effective strategies for Cd/As co-control in rice.

## 2 Materials and methods

### 2.1 Test soil and materials

The test soil (0–20 cm) was collected from the paddy field surrounding the Qibaoshan mining area in Liuyang, Hunan Province, China (28°29′ N, 113°37′ E). The soil was a red soil developed from slate parent material and was co-contaminated with Cd and As, with total Cd and As concentrations of 2.13 and 174.05 mg kg^−1^, respectively. After collection, the soil was air-dried, manually cleaned to remove large stones and plant roots, passed through a 2-mm nylon sieve, thoroughly homogenized, and stored for the pot experiment. FA was provided by Shandong Quanlin Group Co., Ltd. Woody peat (PT) was obtained from Hebei Dewoduo Fertilizer Co., Ltd. HA, representing the humate fractions, was extracted from PT with sodium hydroxide under a controlled solid–liquid ratio. The pH, total organic carbon (TOC), total nitrogen (TN), C/N ratio, total phosphorus (TP), total potassium (TK), and total Cd/As concentrations of the soil and amendments are listed in **Supplementary Table S1**. The rice cultivar used was Xiangwanxian 12, a late indica rice cultivar.

### 2.2 Pot experiment

The pot experiment was conducted during the 2019 late-rice growing season in a greenhouse at the Changsha Agricultural Environment Observation Station of the Chinese Academy of Sciences. Eight treatment combinations were established with two water regimes and four amendment treatments. The two water regimes were flooding–moisture alternation (FM) and continuous flooding (F). The amendment treatments included CK (no amendment), FA, HA, and PT, with FA, HA, and PT each applied at 4 g C kg^−1^ soil under FM and F. Thus, the eight treatments were FM-CK, FM-FA, FM-HA, FM-PT, F-CK, F-FA, F-HA, and F-PT. Each treatment had three replicates, giving a total of 24 pots. All amendments were thoroughly mixed with the soil before the pots were filled. Each plastic pot was filled with 5 kg of treated soil. Basal fertilizers were applied at rates of 200 mg N kg^−1^, 100 mg P_2_O_5_ kg^−1^, and 200 mg K_2_O kg^−1^ using urea, superphosphate, and potassium chloride, respectively. Rice seedlings with uniform growth were transplanted into each pot, with three seedlings per pot. All pots were arranged in a completely randomized design in the greenhouse. The F treatment was maintained under a 3- to 5-cm water layer throughout the rice-growing period, with water added as needed to maintain this depth. Under the FM treatment, the pots were flooded to a water depth of 3–5 cm and then allowed to dry naturally until no visible standing water remained on the soil surface, at which point the soil moisture content was approximately 30%. The pots were subsequently reflooded to a depth of 3–5 cm. This cycle was repeated until sampling at the tillering stage (4 cycles), filling stage (11 cumulative cycles), and maturity stage (20 cumulative cycles). The duration of each FM cycle was not fixed but depended on the time required for the soil to reach the target moisture content.

### 2.3 Sample collection and analysis

Paired soil and rice samples were collected from the same pots at the tillering, filling, and maturity stages. At each stage, one rice plant was destructively collected from each pot, whereas soil samples were repeatedly collected from the same pot. Thus, each pot was considered one experimental unit and one biological replicate (*n* = 3). Rice plants were thoroughly rinsed with deionized water and separated into roots and shoots at tillering, roots, stems, and leaves at filling, and roots, stems, leaves, and brown rice at maturity. Fresh root samples were collected for IP extraction. After extraction, the roots were thoroughly rinsed with deionized water, dried, ground, and subsequently used for tissue Cd/As determination. The remaining plant tissues were partially dehydrated at 105°C for 30 min and then further dried at 65°C for 48 h until reaching a constant weight.

Soil pH, redox potential (Eh), and total and available Cd/As were determined according to Li et al. (2021). The IP-associated metals (Cd_IP_, As_IP_, and Fe_IP_) were extracted using the dithionite–citrate–bicarbonate (DCB) method and quantified following Li et al. (2022). The amounts of Cd_IP_, As_IP_, and Fe_IP_ in the DCB extracts were normalized to the dry weight of the corresponding roots. The Cd/As contents in different rice tissues were determined after microwave-assisted HNO_3_-H_2_O_2_ digestion using inductively coupled plasma mass spectrometry (ICP-MS) according to Li et al. (2022). The Cd and As concentrations determined in rice tissues represented total Cd and total As, respectively. Detailed analytical steps and quality-control information are provided in the Supporting Information.

### 2.4 Calculation and statistical analysis

The concentration-based transfer factors (TFs) of Cd and As at maturity were calculated to describe their relative distribution among rice tissues. The TF_root-stem,_ TF_stem-leaf_, and TF_shoot-grain_ were calculated as follows (Equations 1–3):

(1) $${\text{TF}}_{\text{root-stem}}={\text{C}}_{\text{stem}}/{\text{C}}_{\text{root}}$$

(2) $${\text{TF}}_{\text{stem-leaf}}={\text{C}}_{\text{leaf}}/{\text{C}}_{\text{stem}}$$

(3) $${\text{TF}}_{\text{shoot-grain}}={\text{C}}_{\text{grain}}/{\text{C}}_{\text{shoot}}$$

where C_root_, C_stem_, C_leaf_, and C_grain_ represent Cd/As contents in roots, stems, leaves, and grains at the maturity stage, respectively. C_shoot_ was operationally defined as the arithmetic mean of the Cd or As concentrations in stems and leaves at maturity. Thus, the calculated TFs represent concentration-based transfer indices rather than mass fluxes.

The reduction rates of soil available Cd/As and grain Cd/As were calculated relative to the CK under the same water regimes as follows (Equation 4):

(4) $$\text{Reduction rate}\left(\%\right)=\left[\left({\text{C}}_{\text{CK}}-{\text{C}}_{\text{amendments}}\right)/{\text{C}}_{\text{CK}}\right]\times 100$$

where C_CK_ and C_amendments_ represent the Cd/As concentration in CK and the corresponding amendment treatment, respectively.

The grain Cd/As simultaneous co-reduction index (SCI, defined in this study) was calculated as the lower of the Cd and As reduction rates (Equation 5):

(5) $$\text{SCI}\left(\%\right)=\min\left({\text{R}}_{\text{Cd}},{\text{R}}_{\text{As}}\right)$$

where R_Cd_ and R_As_ represent the reduction rates of grain Cd and grain As, respectively. The SCI was defined using the lower of the Cd and As reduction rates to provide a conservative measure of simultaneous co-reduction and to prevent a decrease in one element from offsetting an increase in the other. Accordingly, a positive SCI indicates simultaneous reductions in both Cd and As, whereas a negative SCI indicates an increase in at least one of the two elements.

The screening-level grain risk index (GRI) was calculated as the sum of the ratios of grain Cd and As concentrations to their corresponding reference values (Equations 6–8):

(6) $$\text{GRI}={\text{E}}_{\text{Cd}}+{\text{E}}_{\text{As}}$$

(7) $${\text{E}}_{\text{Cd}}={\text{C}}_{\text{grain Cd}}/{\text{C}}_{\text{ref Cd}}$$

(8) $${\text{E}}_{\text{As}}={\text{C}}_{\text{grain As}}/{\text{C}}_{\text{ref As}}$$

where C_grain Cd_ and C_grain As_ represent the total Cd and total As contents in brown rice at maturity, respectively. C_ref Cd_ and C_ref As_ were set at 0.2 and 0.35 mg kg^−1^, respectively, according to the National Food Safety Standard GB 2762–2022 (National Health Commission of the People’s Republic of China and State Administration for Market Regulation, 2022). The Cd reference value applies to total Cd, whereas the As reference value applies to inorganic As in brown rice. In this formulation, Cd and As were equally weighted after normalization to their respective reference values, allowing comparison of their combined relative levels among treatments. Because inorganic As was not determined in this study, total grain As was conservatively used as a surrogate in the calculation of EAs. Therefore, GRI was interpreted together with the individual grain Cd/As contents and SCI as a screening-level comparative indicator, rather than as a direct measure of regulatory compliance or absolute food-safety risk.

Statistical analyses were performed using SPSS Statistics (IBM Corp., Armonk, NY, USA). All data are expressed as means ± standard deviation (SD). Two-way analysis of variance (ANOVA) was used to evaluate the effects of the water regime, organic amendment, and their interaction on Cd/As accumulation and translocation in rice tissues, grain Cd/As reduction rates, SCI, and GRI. Linear mixed-effects models were used to evaluate the effects of water regime, organic amendment, rice growth stage, and their interactions on soil properties, available Cd/As, and metals in IP, with pot identity included as a random intercept to account for repeated sampling of the same pots. For simple-effect comparisons, differences among amendment treatments within each water regime and growth stage were evaluated using one-way ANOVA followed by the LSD test at *p* < 0.05. Pearson correlation analysis was used to examine the relationship among all measured parameters. Linear regression analyses were conducted separately for each water regime using maturity-stage pot-level observations to evaluate the relationships among soil Cd/As availability, IP-associated metals, and Cd/As concentrations in rice tissues. Redundancy analysis (RDA) was conducted using the 24 pot-level observations to identify the main factors associated with grain Cd/As accumulation and risk indicators. Multicollinearity among explanatory variables was assessed using variance inflation factors (VIFs), and variables with VIF > 10 were sequentially removed. The significance of the overall RDA model was evaluated using 999 permutations, and the adjusted *R*^2^ was calculated to assess the explanatory power of the final model.

## 3 Results

### 3.1 Changes in soil pH, Eh, and Cd/As availability during key rice growth

Linear mixed-effects models showed that the soil pH, Eh, and available Cd/As were significantly affected by water regime, organic amendment, and rice growth stage (*p* < 0.001, **Table 1**). Significant water regime × amendment × growth stage interactions were observed for soil pH and available Cd/As, whereas the corresponding interaction was not significant for Eh.

**Table 1:** Linear mixed-effects model results for soil properties, Cd/As availability, and metals in iron plaque.

Variable	Water (W), *F*(1, 16)	Amendment (A), *F*(3, 16)	Stage (S), *F*(2, 32)	W×A, *F*(3, 16)	W×S, *F*(2, 32)	A×S, *F*(6, 32)	W×A×S, *F*(6, 32)
pH	50.18***	69.18***	115.64***	5.11*	4.74*	19.43***	4.26**
Eh	601.39***	11.38***	20.57***	1.88 ns	138.24***	1.70 ns	0.66 ns
CaCl_2_-Cd	2,433.78***	292.96***	454.23***	182.28***	223.37***	109.80***	100.87***
KH_2_PO_4_-As	1,238.51***	32.42***	162.82***	29.77***	304.36***	9.37***	8.25***
Cd_IP_	459.20***	38.42***	12.13***	20.04***	161.18***	13.24***	11.54***
As_IP_	877.59***	30.69***	5,270.55***	230.95***	1,549.02***	100.02***	77.46***
Fe_IP_	0.02 ns	69.84***	359.79***	75.45***	63.82***	90.70***	55.85***
Cd_IP_/Fe_IP_	541.15***	4.98**	117.90***	4.27**	230.04***	13.79***	12.69***
As_IP_/Fe_IP_	266.97***	24.86***	220.77***	86.81***	115.45***	39.77***	25.42***

Values are *F*-statistics from the Type III tests of fixed effects. Numerator and denominator degrees of freedom are shown in the column headings. Pot identity was included as a random intercept. Cd_IP_, As_IP_, and Fe_IP_ indicate Cd, As, and Fe in iron plaque, respectively; ***p < 0.001, **p < 0.01, *p < 0.05, ns, not significant (*p* ≥ 0.05).

The soil pH in all amendments generally declined under the two water regimes (**Figure 1a**). Relative to the CK, soil pH in the HA significantly increased by 0.42–1.19 units and 0.24–0.68 units across rice growth stages under FM and F, respectively, whereas soil pH in the FA markedly decreased by 0.62–0.97 units and 0.26–0.49 units at the filling and maturity stages under FM and F (*p* < 0.05, **Supplementary Figures S1a, b**), respectively. Notably, PT showed only slight differences from CK under FM and F. The soil Eh exhibited distinct responses to water management practices, with an increase under FM and a decline under F across rice growth stages (**Figure 1b**). Relative to the CK, soil Eh in all treatments showed no significant difference under FM. In contrast, soil Eh in the FA decreased by 93.10–109.20 mV at the filling and maturity stages under F, while soil Eh in the HA decreased by 61.27 mV at the maturity stage (*p* < 0.05, **Supplementary Figures S1c, d**). Notably, the soil pH values were higher under F than under FM, while soil Eh was lower under F than under FM.

**Figure 1:**
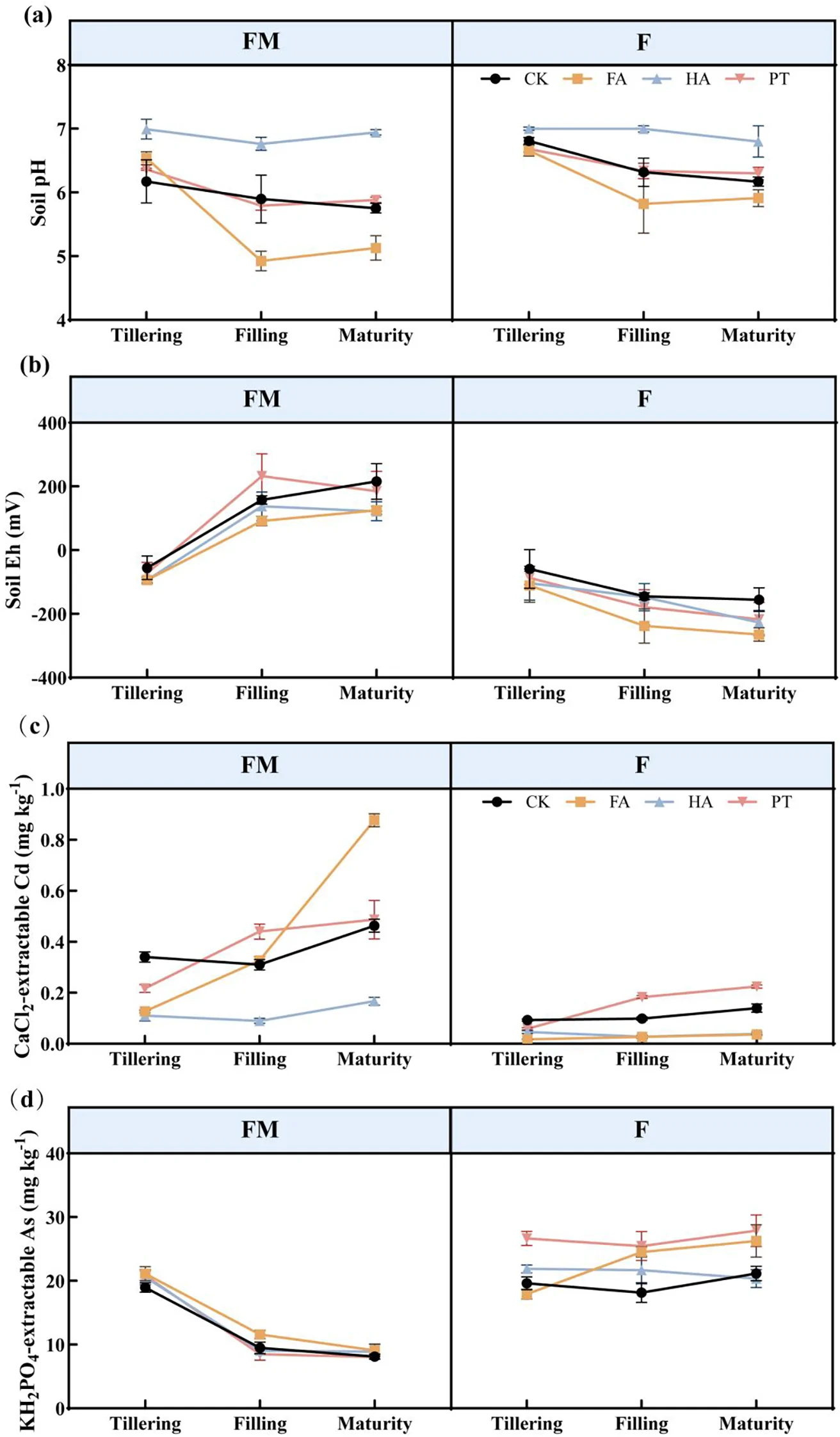
Changes in soil pH **(a)**, Eh **(b)**, CaCl_2_-extractable Cd **(c)**, and KH_2_PO_4_-extractable As **(d)** under different water regimes and organic amendments during key rice growth stages. FM, flooding–moisture alternation; F, continuous flooding. Values are means ± SD (*n* = 3). The error bars represent the SDs. Within each water regime and growth stage, differences among amendment treatments were compared using one-way ANOVA followed by the LSD test at a probability level of 0.05, and the corresponding significance labels are provided in **Supplementary Figure S1**.

The soil Cd/As availability showed distinct responses to organic amendment type and water management practices (**Figures 1c, d**). CaCl_2_-Cd in all treatments generally increased across rice growth stages under the two water regimes, but the magnitude and treatment effects markedly differed (**Supplementary Figures S1e, f**). CaCl_2_-Cd in the FA and PT reduced at the tillering stage but showed increased Cd availability at later stages, especially in the FA at maturity. In contrast, CaCl_2_-Cd in the FA and HA maintained a low Cd availability under F across rice growth stages, whereas the PT resulted in higher Cd availability, particularly at the filling and maturity stages. However, KH_2_PO_4_-As exhibited different trends under the two water regimes. Under FM conditions, KH_2_PO_4_-As in all treatments declined across rice growth stages, with no significant differences among treatments (**Supplementary Figure S1g**). Under F conditions, the KH_2_PO_4_-As in the FA was lower than that in the CK at the tillering stage, but higher than the CK at the filling and maturity stages (**Supplementary Figure S1h**). KH_2_PO_4_-As in the PT was consistently higher than that in the CK across rice growth stages, whereas KH_2_PO_4_-As in the HA was higher than the CK at the tillering and filling stages.

The reduction rates further confirmed the contrasting responses of soil Cd/As availability to organic amendments (**Supplementary Figure S2**). For available Cd, the HA exhibited stable positive reduction rates across rice growth stages, with reductions of 63.88%–71.51% under FM and 50.33%–71.81% under F (*p* < 0.05, **Supplementary Figures S2a, b**). The FA decreased CaCl_2_-Cd by 72.02%–81.48% under F across rice growth stages, whereas it reduced CaCl_2_-Cd by 63.07% only at the tillering stage under FM (*p* < 0.05). The PT decreased CaCl_2_-Cd only at the tillering stage under FM and F, with reduction rates of 37.55% and 36.68%, respectively, while increased Cd availability was observed at later stages (*p* < 0.05). For available As, the reduction rates were closest to zero under FM, indicating limited effects of organic amendments on KH_2_PO_4_-As (**Supplementary Figures S2c, d**). In contrast, all amendments increased KH_2_PO_4_-As under F, especially at the later stages. The FA increased KH_2_PO_4_-As by 24.22%–35.75% at the filling and maturity stages, while PT increased KH_2_PO_4_-As by 31.85%–40.99% across rice growth stages (*p* < 0.05). Notably, the HA showed a comparatively weaker As-mobilizing effect, with changes close to CK or only slightly higher.

### 3.2 Changes in Fe, Cd, and As concentrations in iron plaque during rice growth

Linear mixed-effects models showed that the Cd_IP_ and As_IP_ were significantly affected by water regime, organic amendment, rice growth stage, and their interactions (*p* < 0.001, **Table 1**). Fe_IP_ was significantly affected by organic amendments, rice growth stage, and their interactions (*p* < 0.001, **Table 1**), whereas the main effect of water regime on Fe_IP_ was not significant. The Cd_IP_/Fe_IP_ and As_IP_/Fe_IP_ ratios were also significantly affected by water regime, organic amendment, rice growth stage, and their interactions (*p* < 0.01, **Table 1**).

The Fe_IP_ in the CK, HA, and PT usually declined across rice growth stages under the two water regimes, whereas Fe_IP_ in FA showed stage-dependent changes (**Figure 2a**). Relative to the CK, Fe_IP_ in the HA significantly decreased by 13.35%–52.41% under FM across rice stages, whereas Fe_IP_ in the PT decreased by 11.27%–41.42% at the tillering and filling stages (*p* < 0.05, **Supplementary Figure S3a**). Notably, Fe_IP_ in the FA was lower (22.47%) than that in the CK at the tillering stage but increased markedly (131.42%) at the maturity stage (*p* < 0.05). Relative to the CK, Fe_IP_ in the PT increased by 24.52% at tillering, that in the HA increased by 23.10% at the filling stage, and that in the FA increased by 29.51% at the maturity stage, whereas the Fe_IP_ in the FA at the tillering stage and in the HA/PT at the maturity stage decreased by 23.68%–40.57% (*p* < 0.05, **Supplementary Figure S3b**).

**Figure 2:**
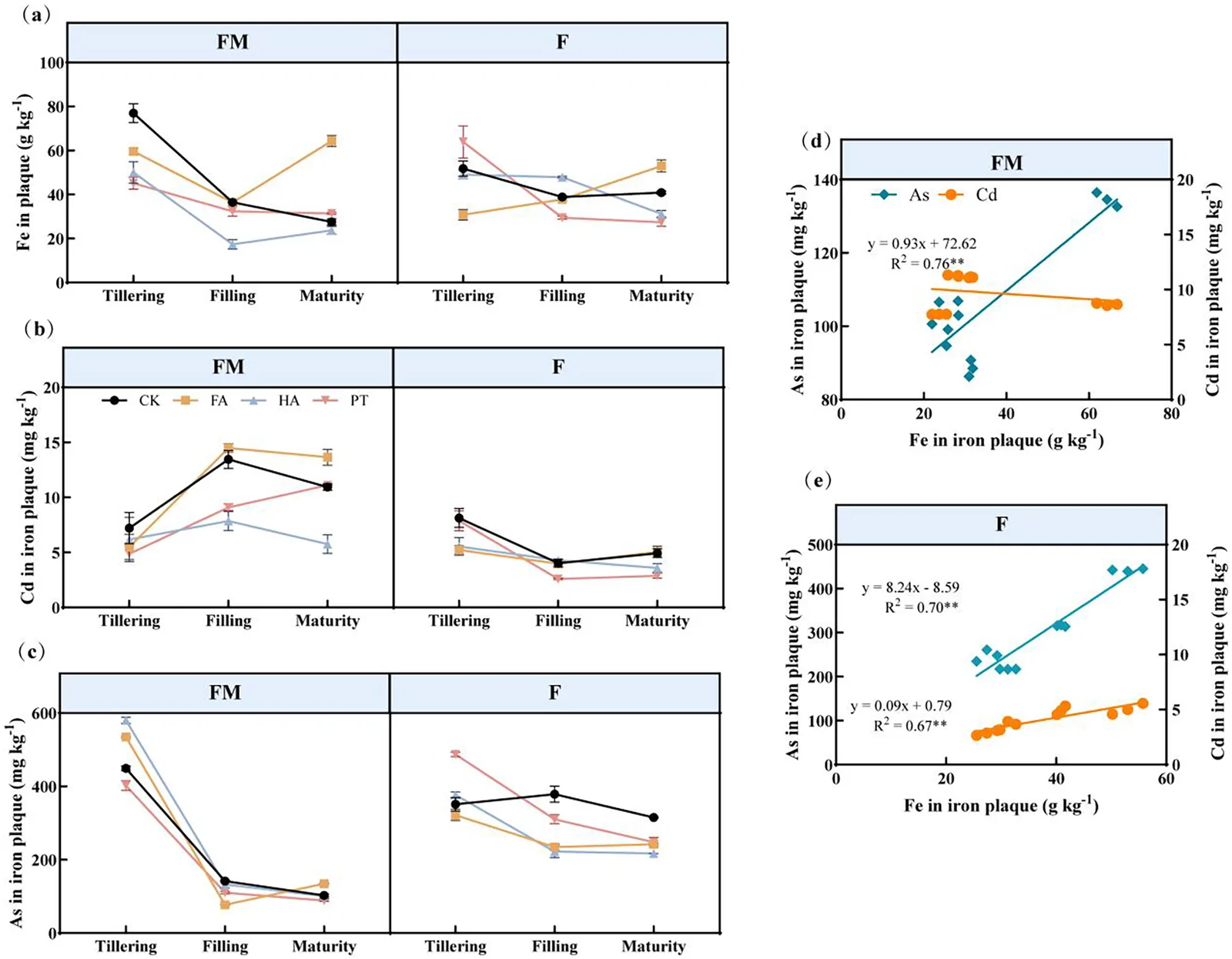
Changes in Fe, Cd, and As concentrations in iron plaque (FeIP, CdIP, and AsIP) and their relationships under different water regimes and organic amendments during key rice growth stages. **(a)** FeIP, **(b)** CdIP, and **(c)** AsIP. Relationships between FeIP and CdIP/AsIP under **(d)** FM and **(e)** F conditions. FM, flooding–moisture alternation; F, continuous flooding. Values are means ± SD (*n* = 3). The error bars represent the SDs. Within each water regime and growth stage, differences among amendment treatments were compared using one-way ANOVA followed by the LSD test at a probability level of 0.05, and the corresponding significance labels are provided in **Supplementary Figure S3**. For panels **(d, e)**, solid lines indicate fitted linear regressions, and *R*^2^ and *p*-values indicate the goodness of fit and statistical significance of the regression, respectively.

The Cd_IP_ in all treatments increased from tillering to the filling stage under FM, whereas it generally declined from tillering to the filling stage under F and remained low at the maturity stage (**Figure 2b**). Relative to the CK, Cd_IP_ in the HA decreased by 41.39%–47.42% at the filling and maturity stages under FM, whereas Cd_IP_ in the PT decreased by 32.65% at the filling stage (*p* < 0.05, **Supplementary Figure S3c**). In contrast, Cd_IP_ in the FA increased by 24.93% at the maturity stage under FM. Notably, Cd_IP_ in all treatments showed no significant difference at the tillering stage under FM. Relative to the CK, Cd_IP_ in the FA and HA decreased by 32.50% and 30.34% at the tillering stage under F, respectively, whereas Cd_IP_ in the PT decreased by 35.25%–41.72% at the filling and maturity stages (*p* < 0.05, **Supplementary Figure S3d**). The Cd_IP_ showed a positive relationship with Fe_IP_ under F (*y* = 0.09*x* + 0.79, *R*^2^ = 0.67, *p* < 0.01).

The As_IP_ in all treatments generally declined across rice growth stages under the two water regimes (**Figure 2c**). Relative to the CK, As_IP_ in the PT decreased by 10.46%–22.32% across rice growth stages under FM, whereas As_IP_ in the FA decreased by 45.64% only at the filling stage (*p* < 0.05, **Supplementary Figure S3e**). In contrast, As_IP_ in the FA increased by 19.00% and 30.82% at the tillering and maturity stages under FM, respectively, whereas As_IP_ in the HA increased by 29.11% only at the tillering stage. Relative to the CK, As_IP_ in the FA decreased by 8.04%–37.91% across rice growth stages under F, whereas As_IP_ in the HA and PT decreased by 7.37% and 38.99% at the tillering stage, respectively, but increased As_IP_ by 17.78%–41.31% at the filling and maturity stages (*p* < 0.05, **Supplementary Figure S3f**). The As_IP_ showed a positive relationship with Fe_IP_ under FM (*y* = 0.93*x* + 72.62, *R*^2^ = 0.76, *p* < 0.01) and F (*y* = 8.24*x* − 8.59, *R*^2^ = 0.70, *p* < 0.01).

The Cd_IP_/Fe_IP_ and As_IP_/Fe_IP_ ratios showed contrasting temporal patterns under the two water regimes (**Supplementary Figure S4**). Under FM, Cd_IP_/Fe_IP_ generally increased from tillering to filling, whereas As_IP_/Fe_IP_ declined across rice growth stages. Under F, Cd_IP_/Fe_IP_ decreased from tillering to filling and then increased slightly at maturity, while As_IP_/Fe_IP_ showed amendment-dependent changes.

### 3.3 Cd and As accumulation and translocation in rice tissues at maturity

Two-way ANOVA showed that most Cd/As concentrations in mature rice tissues and their translocation factors were significantly affected by the water regime, organic amendment, and their interactions (*p* < 0.01, **Supplementary Table S2**). Significant W × A interactions were observed for most variables, except for stem Cd and leaf As. In addition, Cd/As concentrations in rice tissues at the tillering and filling stages were also significantly influenced by the water regime, organic amendment, and their interaction effects (*p* < 0.01, **Supplementary Table S3**).

The Cd contents differed significantly among rice tissues at maturity (**Figures 3a, b**). The Cd mainly accumulated in stems and leaves, followed by roots and grains under the two water regimes. Relative to the CK, the HA addition reduced grain, leaf, stem, and root Cd by 13.56%, 45.44%, 20.34%, and 30.51% under FM, respectively, and by 66.88%, 59.09%, 72.36%, and 41.32% under F, respectively. The FA reduced leaf, stem, and root Cd by 36.89%, 24.21%, and 25.79% under FM, respectively, but had no significant effect on grain Cd. Similarly, the FA reduced grain, leaf, and stem Cd by 34.51%, 31.37%, and 56.30% under F, respectively. Under FM, PT reduced grain, stem, and root Cd concentrations by 47.20%, 10.75%, and 53.05%, respectively. Under F, PT reduced grain, leaf, stem, and root Cd concentrations by 60.08%, 52.72%, 66.70%, and 49.23%, respectively. Different organic amendment additions revealed that the PT achieved the greatest reduction in grain Cd under FM, whereas HA achieved the greatest reduction under F.

**Figure 3:**
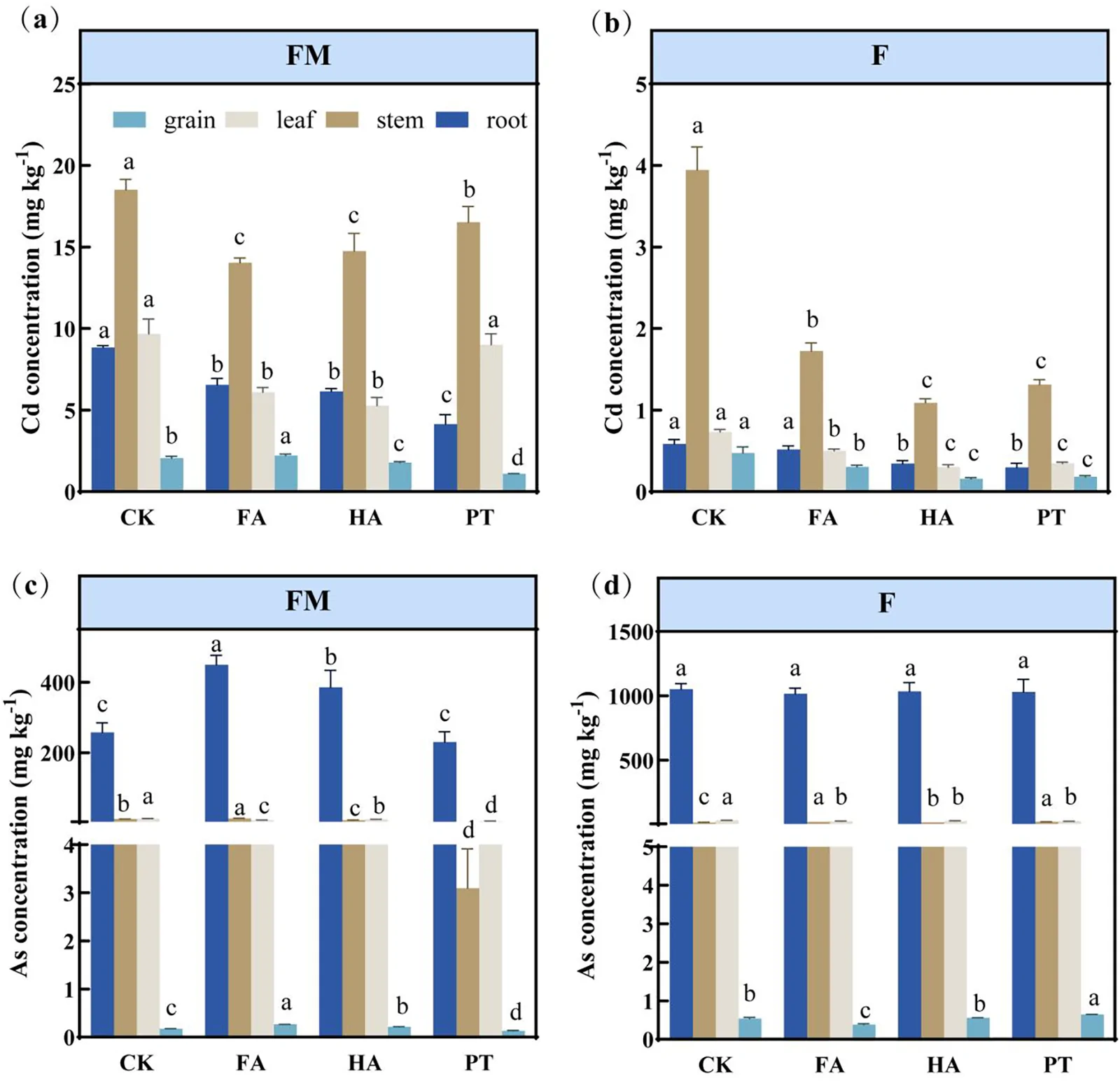
Cd and As contents in rice tissues (grains, leaves, stems, and roots) under different water regimes and organic amendments at the maturity stage. **(a, b)** Cd contents in rice tissues under FM and F, respectively. **(c, d)** As contents in rice tissues under FM and F, respectively. FM, flooding–moisture alternation; F, continuous flooding. Values are means ± SD (*n* = 3). The error bars represent the SDs. Different lowercase letters indicate significant differences among treatments at the same sampling time using a one-way ANOVA followed by the LSD test at a probability level of 0.05.

Unlike Cd, the As contents showed a different distribution in rice tissues (**Figures 3c, d**). Root As contents were substantially higher than those in stems, leaves, and grains under the two water regimes. Relative to the CK, FA and HA addition increased root As by 74.51% and 49.79% under FM, respectively, whereas PT slightly decreased root As. In contrast, PT showed the lowest grain As and reduced it by 23.96% under FM, whereas FA and HA increased grain As by 53.66% and 25.63%. Notably, root As under F was higher than that under FM, but no significant differences were observed among treatments. In contrast, grain As under F was generally higher than that under FM. Relative to the CK, PT addition increased grain As by 20.39%, and FA decreased grain As by 28.44%, whereas HA caused a slight increase in grain As. Different organic amendment additions revealed that the PT achieved the greatest reduction in grain As under FM, whereas FA achieved the greatest reduction under F.

The translocation factors further revealed the effects of amendments and water regimes on grain Cd/As (**Supplementary Figure S5**). Relative to the CK, PT increased TF_root-stem_ of Cd but decreased TF_shoot-grain_ of Cd under FM, whereas FA and HA increased TF_shoot-grain_ of Cd but decreased TF_stem-leaf_ of Cd under FM. All amendments decreased TF_root-stem_ of Cd under F but enhanced TF_stem-leaf_ of Cd relative to the CK. Notably, FA increased TF_shoot-grain_ of Cd under F, while HA and PT showed no significant differences from CK. Relative to the CK, all amendments decreased TF_root-stem_ of As under FM. PT increased both TF_stem-leaf_ and TF_shoot-grain_ of As, whereas FA and HA only increased TF_shoot-grain_ of As. In contrast, all amendments decreased TF_stem-leaf_ of As under F relative to the CK. PT increased both TF_root-stem_ and TF_shoot-grain_ of As, whereas FA increased TF_root-stem_ and decreased TF_shoot-grain_ of As. HA only increased TF_shoot-grain_ of As.

The Cd/As accumulation in rice tissues showed stage-dependent changes during rice growth (**Supplementary Figure S6**). Root Cd under FM first increased from tillering to filling stage and then declined at the maturity stage, whereas stem and leaf Cd gradually increased during rice growth. Notably, Cd contents in roots, stems, and leaves under F were lower than those under FM. In contrast, As contents in roots, stems, and leaves under F were much higher than those under FM. Root As gradually decreased during rice growth under FM, while root As showed treatment-dependent trends. Stem As showed a decline from tillering to filling stage and an increase at the maturity stage under both water regimes, whereas leaf As increased from the filling to maturity stage.

### 3.4 Grain Cd/As reduction and risk assessment

Two-way ANOVA showed that the grain Cd/As reduction rate, grain Cd/As SCI, and grain Cd/As risk index (GRI) were significantly affected by water regime, organic amendment, and their interactions (*p* < 0.001, **Supplementary Table S2**).

Grain Cd/As reduction with different amendments differed markedly between the two water regimes (**Figures 4a, b**). Under FM, the PT simultaneously reduced grain Cd and grain As, with reduction rates of 47.20% and 23.96%, respectively. In contrast, the HA reduced grain Cd by 13.56% but increased grain As by 25.63%, whereas the FA slightly increased grain Cd by 7.18% and increased grain As by 53.66%. Under F, all amendments reduced grain Cd by 34.51%–66.88%, but the effects on grain As differed among amendments. The FA reduced grain As by 28.44%, whereas PT increased grain As by 20.39%, and the HA showed only a weak effect.

**Figure 4:**
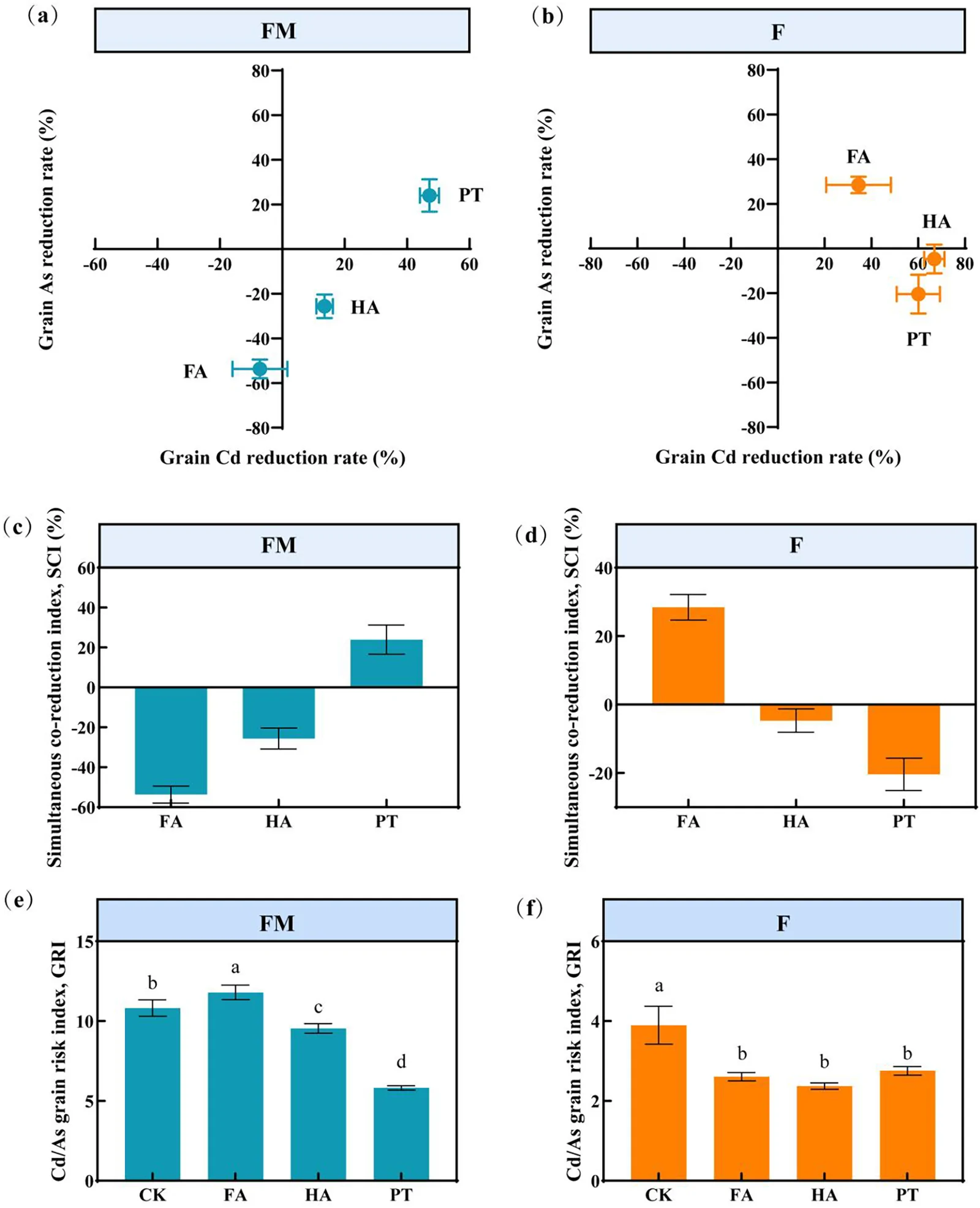
Grain Cd/As reduction, co-reduction index, and grain risk index under different water regimes and organic amendments. **(a, b)** Relationships between grain Cd reduction rate and grain As reduction rate under FM and F conditions, respectively. **(c, d)** Grain Cd/As simultaneous co-reduction index (SCI) under FM and F conditions, respectively. **(e, f)** Screening-level grain risk index (GRI) under FM and F conditions, respectively. Values are means ± SD (*n* = 3). The error bars represent the SDs. FM, flooding–moisture alternation; F, continuous flooding. Positive reduction values indicate decreases relative to the corresponding CK under the same water regime, whereas negative values indicate increases. For SCI, positive values indicate simultaneous reductions in grain Cd and As, whereas negative values indicate an increase in at least one element. Different lowercase letters indicate significant differences among treatments at *p* < 0.05.

The SCI further distinguished simultaneous Cd/As reduction from trade-off responses between the two elements (**Figures 4c, d**). Under FM, only PT showed a positive SCI (23.96%), whereas FA and HA showed negative SCI values of −53.66% and −25.63%, respectively. Under F, only FA showed a positive SCI (28.44%), whereas HA and PT showed negative SCI values of −4.69% and −20.39%, respectively. These results indicate that simultaneous reductions in grain Cd and As were achieved by PT under FM and by FA under F.

The changes in GRI generally corresponded to the grain Cd/As co-reduction performance under the two water regimes (**Figures 4e, f**). Relative to the CK, the FA increased the GRI value by 9.03% under FM, whereas HA and PT decreased the GRI values by 11.84% and 46.24%, respectively. Relative to the CK, all amendments decreased GRI values by 29.29%–39.17% under F, with HA showing the lowest grain risk. Among the organic amendments, the PT showed the lowest grain Cd/As risk under FM, while HA achieved the strongest risk reduction under F.

### 3.5 Associations of soil and plant variables with grain Cd/As accumulation and risk

Correlation analysis showed that the relationships among soil properties, Cd/As availability, metals in IP, Cd/As translocation factors, and grain risk indicators differed significantly between the two water regimes (**Figure 5**). Under FM, grain Cd was closely correlated with root Cd and TF_shoot-grain_ of Cd, while grain As was strongly associated with stem As, As_IP,_ Fe_IP_, available As, and soil Eh. SCI was negatively correlated with grain Cd, grain As, and GRI. In addition, available Cd was closely related to soil pH, while available As was closely associated with soil Eh. Under F, grain Cd was closely correlated with Cd in other rice tissues and Cd_IP_, while grain As was strongly associated with root As and TF_shoot-grain_ of As. SCI was negatively correlated with grain Cd and GRI. Regression analysis further examined selected relationships identified by the correlation analysis (**Figure 6**). CaCl_2_-Cd showed weak relationships with root Cd under both FM and F (*R*^2^ = 0.008 and 0.074, respectively), indicating that CaCl_2_-Cd alone explained little of the variation in root Cd. In contrast, KH_2_PO_4_-As was significantly related to root As, especially under FM (*R*^2^ = 0.492 under FM and 0.291 under F; **Figure 6**). Furthermore, Cd_IP_ was significantly associated with grain Cd and root Cd only under F, while As_IP_ was significantly associated with grain As and root As only under FM. To further integrate these relationships, RDA was conducted with soil pH, Eh, Cd/As availability, metals in IP, and TF_shoot-grain_ of Cd/As explanatory variables, and grain Cd/As, SCI, and GRI as response variables (**Figure 7**). After VIF screening, six explanatory variables were retained, namely, available Cd, As_IP_, Fe_IP_, pH, TF_shoot-grain_ of Cd, and TF_shoot-grain_ of As. Their VIF values were 6.94, 3.38, 4.38, 6.70, 3.36, and 1.62, respectively. The RDA model was significant (*F* = 14.04, df = 6, 17, *p* = 0.001), with an adjusted *R*^2^ of 0.78. The first two RDA axes explained 74.31% and 10.65% of the ordination variation, respectively. Grain Cd, grain As, SCI, and GRI showed different positions along the ordination axes and were associated with different explanatory variables. Given the limited sample size, the RDA was interpreted as an exploratory rather than confirmatory analysis.

**Figure 5:**
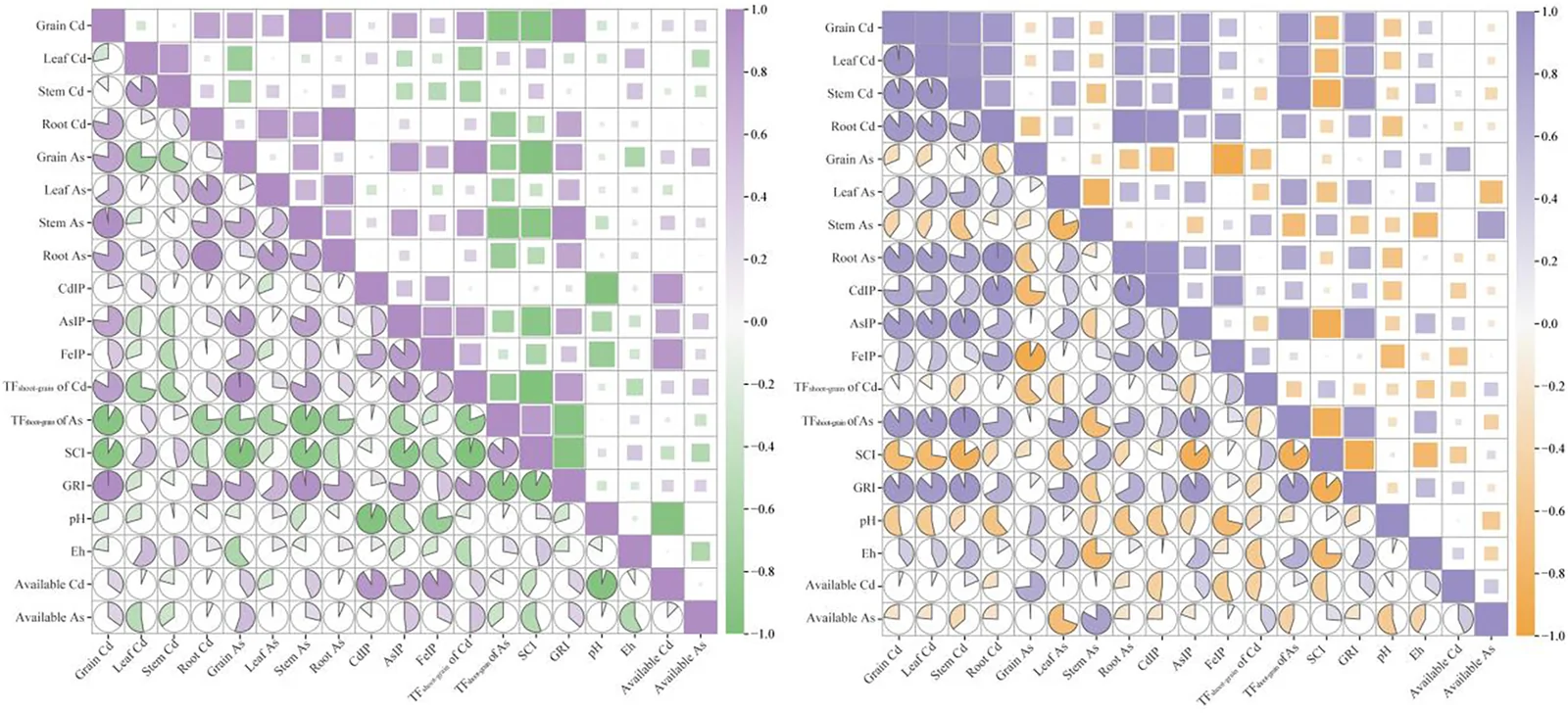
Relationships among soil properties, available Cd/As, metals in iron plaque, rice Cd/As accumulation, translocation factors, and grain risk indices under FM **(a)** and F **(b)** conditions at the maturity stage (*n* = 12).

**Figure 6:**
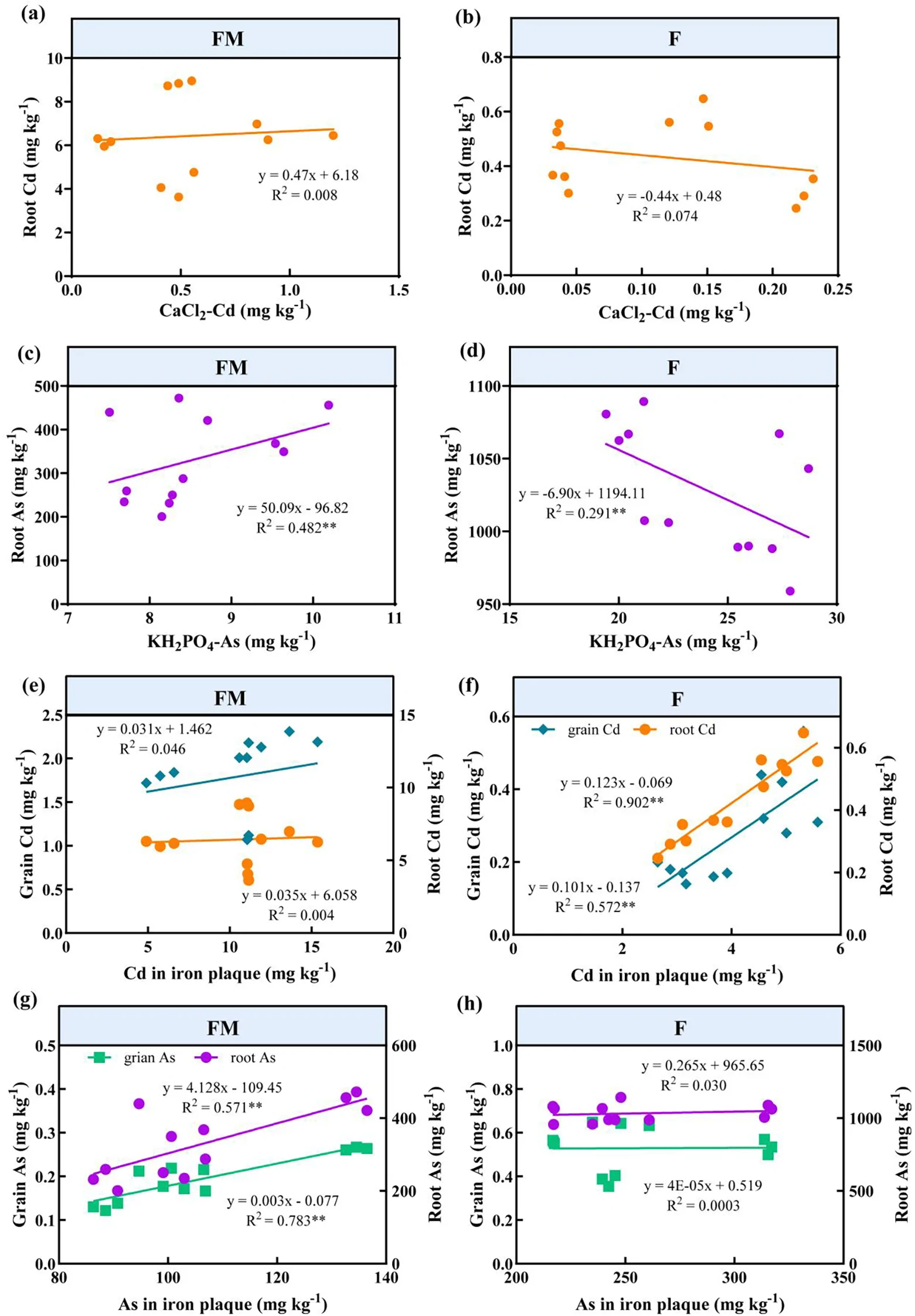
Key regression relationships among soil Cd/As availability, Cd/As in iron plaque, and plant Cd/As accumulation under different water regimes. **(a, b)** Relationships between CaCl_2_-Cd and root Cd, **(c, d)** relationships between KH_2_PO_4_-As and root As, **(e, f)** relationships between Cd in iron plaque and root/grain Cd, and **(g, h)** relationships between As in iron plaque and root/grain As under FM and F. Each point represents one maturity-stage pot-level observation (*n* = 12 per water regime). Solid lines indicate fitted linear regressions. *R*^2^ and significance levels indicate the goodness of fit and statistical significance of the regressions. FM, flooding–moisture alternation; F, continuous flooding. *p < 0.05; **p < 0.01.

**Figure 7:**
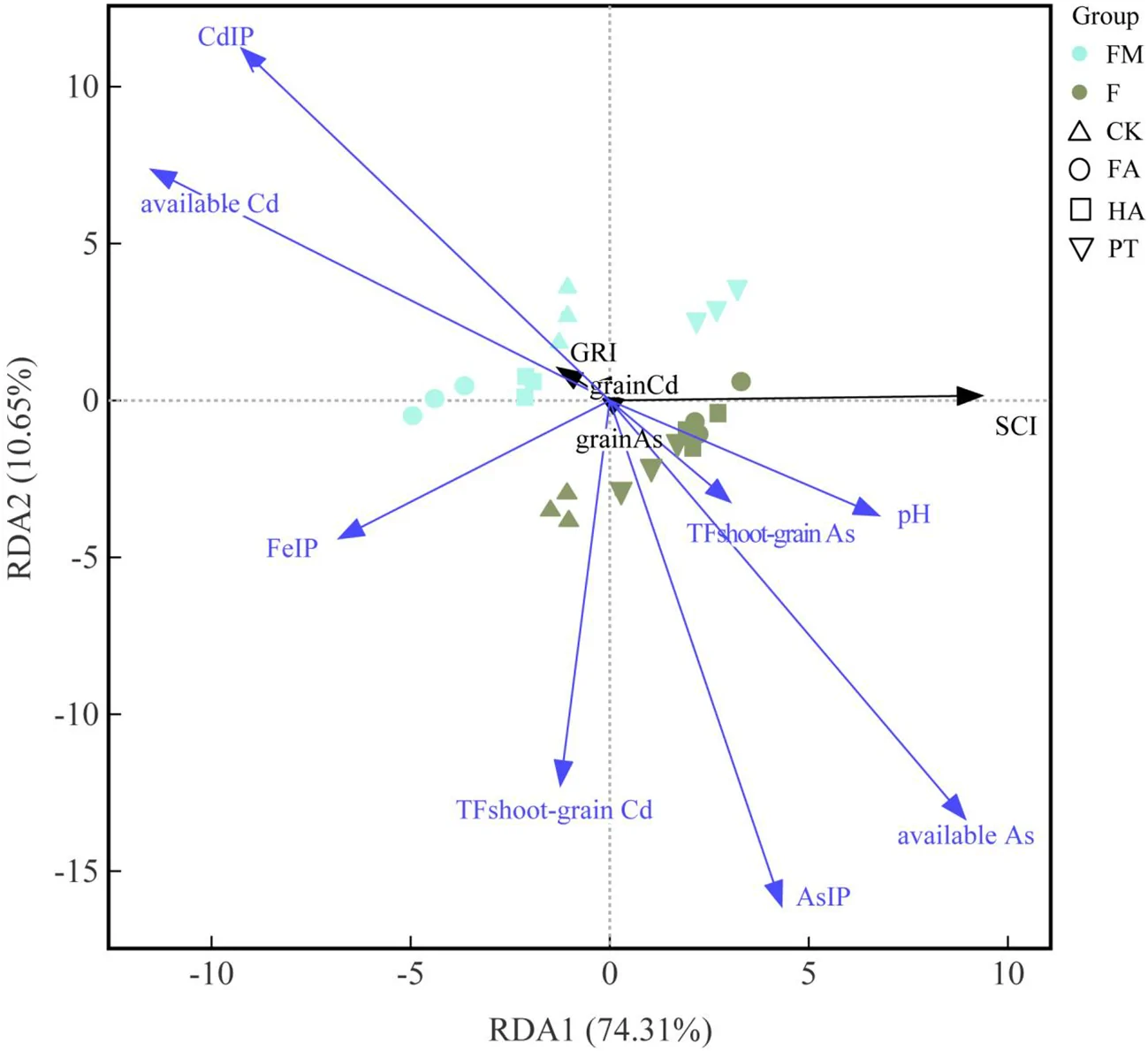
Redundancy analysis (RDA) of grain Cd/As accumulation, the simultaneous co-reduction index (SCI), and the grain risk index (GRI) at maturity. After VIF screening, the six retained explanatory variables were CaCl_2_-Cd, As_IP_, Fe_IP_, soil pH, TF_shoot-grain_ of Cd, and TF_shoot-grain_ of As.

Correlation, regression, and RDA results showed that the factors associated with grain Cd/As accumulation differed between the two water regimes, with soil Cd/As availability, IP-associated metals, and internal translocation showing different relative contributions.

## 4 Discussion

### 4.1 Water regime-dependent effects of organic amendments on soil Cd/As availability

Soil pH and Eh are two key factors regulating Cd/As availability in paddy soils. In this study, the water regime and organic amendments significantly altered soil pH and Eh during rice growth, which was accompanied by distinct changes in Cd/As availability (**Figure 1**). Correlation analysis showed that CaCl_2_-Cd was closely associated with soil pH and KH_2_PO_4_-As was closely associated with Eh under FM, but these relationships were not significant under F (**Figure 5**). These results indicated that the regulation of Cd/As availability by organic amendments depended on the water regime. Although FA, HA, and PT were applied at the same carbon rate, their contrasting effects likely reflected differences in their physicochemical properties (**Supplementary Table S1**).

The closer association between CaCl_2_-Cd and soil pH under flooding–moisture alternation conditions suggests that pH-related processes were associated with variation in Cd availability. Accordingly, the stronger Cd immobilization observed with HA may be primarily attributed to its greater ability to increase soil pH, thereby reducing Cd mobility (Huang et al., 2021; Li et al., 2025). Furthermore, HA may contain oxygen-containing functional groups that could also provide relatively stable Cd-binding sites and further contribute to Cd immobilization (Bai et al., 2018). In contrast, the weaker immobilization with FA may be related to its lower molecular weight, higher solubility, and a relatively acidic pH (5.43), which may have promoted Cd mobilization by lowering soil pH and forming soluble Cd–organic complexes (Li et al., 2021; Jin et al., 2023). The effect of PT on Cd availability may be associated with the decomposition of peat-derived organic matter, which could provide solid organic surfaces for Cd adsorption while also releasing dissolved organic ligands that enhance Cd mobility (Egene et al., 2018). However, this contrast among amendments became less evident under F. The lower Cd availability under continuous flooding is consistent with mechanisms previously reported for reducing paddy soils, including Cd precipitation (e.g., CdS) and adsorption or co-precipitation with Fe-associated phases (Zhang et al., 2022a; Gao et al., 2023). However, these processes were not directly determined in the present study.

The significant association between KH_2_PO_4_-As and Eh under FM suggests that variation in As availability was associated with soil redox conditions. The flooding–moisture alternation-induced low As availability may be related to the redox fluctuations that limited sustained reducing conditions and then restricted the release of soil-bound As, particularly the Fe mineral-associated As pool (Xu et al., 2017; Cheraghi et al., 2025). Furthermore, the effectiveness of the amendment under FM mainly depended on the extent to which each amendment modified mobility near the rhizosphere. FA, with higher solubility, may release soluble organic ligands that enhance As mobilization by competing with As for adsorption sites or by promoting the release and transport of Fe-bound As (Li et al., 2026). In contrast, the weaker effect of HA on As availability may be due to its alkaline nature and lower mobility, which could limit As mobilization (Zhang et al., 2023). PT may also increase As availability through the decomposition of peat-derived organic matter, which may supply labile organic ligands and create more reducing microenvironments (de Oliveira et al., 2015; Aguilar-Garrido et al., 2020). The increased As availability under continuous flooding is consistent with previously proposed redox-related mechanisms, including reductive dissolution of As-bearing Fe minerals and transformation of As(V) to the more mobile As(III) (Honma et al., 2016; Qiao et al., 2019). However, Fe mineralogy, Fe redox transformations, and As speciation were not determined in this study, and these mechanisms therefore remain hypotheses for explaining the observed patterns. The responses of different organic amendments under persistent reduction conditions were increasingly superimposed on the stronger influence of continuous reduction (Cui et al., 2023). Therefore, organic amendments showed water regime-dependent effects on Cd/As availability. Stronger amendment-specific responses were evident under FM, whereas continuous flooding was associated with lower Cd availability but higher As availability under F.

### 4.2 Water regime-dependent effects of organic amendments on iron plaque formation and Cd/As retention

IP is an important soil–root interface regulating Cd/As retention prior to their uptake by rice roots. The IP formation generally depends on the supply of soluble Fe in the rhizosphere and the oxidation of Fe on the root surface (Yuan et al., 2026). In this study, the water regime and organic amendments markedly affected Fe_IP_ during rice growth (**Figure 2**). Flooding–moisture alternation may frequently induce Fe reduction, migration, and re-oxidation in paddy soils and may alter Fe redistribution and precipitation on root surfaces, but such redox fluctuations may not favor sustained Fe_IP_ accumulation (Wang et al., 2022; Zandi et al., 2023). Under such conditions, the increase in Fe_IP_ induced by FA may be related to its high solubility and mobility, which could promote Fe migration and IP formation (Li et al., 2022). However, the lower Fe_IP_ under HA may be related to the complexation of Fe by humic substances, which could reduce Fe precipitation on root surfaces (Li et al., 2020). The stage-dependent effects of PT may be associated with the gradual decomposition of peat-derived organic matter and its influence on Fe reduction and migration during rice growth (Egene et al., 2018). Continuous flooding may promote Fe oxide reduction and increase the supply of soluble Fe(II) to the rhizosphere (Kong et al., 2024), whereas sustained reducing conditions may also constrain Fe oxidation and IP formation on the root surface (Wang et al., 2022). The stage-dependent responses of Fe_IP_ with different organic amendments were consistent with literature-reported effects of organic amendments on Fe mobilization, complexation, and precipitation at the root surface (Li et al., 2022; Chen et al., 2025). More soluble amendments may facilitate Fe redistribution toward the rhizosphere, whereas humic substances and decomposing peat-derived organic matter may also complex Fe and limit its precipitation on root surfaces (Liu et al., 2021).

Both flooding–moisture alternation and continuous flooding altered Cd/As behavior at the soil–root interface, but through different redox-driven pathways (Yin et al., 2026). The IP on rice roots could provide reactive sites for Cd/As adsorption, complexation, and precipitation (Yu et al., 2024; Long et al., 2026). In this study, the water regime and organic amendments markedly affected Cd_IP_ and As_IP_ during rice growth (**Figure 2**). Meanwhile, the Cd_IP_ and As_IP_ showed different relationships with Fe_IP_ (**Figures 2d, e**). The Cd_IP_ was positively associated with Fe_IP_ only under F, whereas the As_IP_ was positively associated with Fe_IP_ under both FM and F (**Figures 2d, e**). For Cd, the relatively higher Cd mobility under flooding–moisture alternation may have increased Cd supply to the soil–root interface and partly compensated for the decrease in IP formation (Wang et al., 2022; Yin et al., 2026), which could explain the weak relationship between Cd_IP_ and Fe_IP_. Furthermore, the amendment effects on Cd_IP_ under FM were likely associated with their regulation of Cd availability and Cd loading on IP. The decreased Cd_IP_ with HA was consistent with its reduction of Cd availability (**Figures 1**, **2**). In contrast, the different responses of Cd_IP_ to PT and FA suggest that Cd retention was influenced by the stage-specific Cd supply to the soil–root interface (**Figure 1**), rather than being simply determined by Fe_IP_ accumulation. Continuous flooding-induced low Cd availability under a reducing environment could limit Cd transport to the soil–root interface and weaken Cd retention on IP (Kong et al., 2024). This may partly explain the lower Cd_IP_ and the closer association between Cd_IP_ and Fe_IP_ under F (**Figure 2d**). The decreases in Cd_IP_ observed with FA, HA, and PT further suggest that amendment effects on Cd_IP_ varied with growth stage under persistent reduction conditions. For As, given the strong affinity of As for Fe-bearing plaque materials (Zandi et al., 2023), As_IP_ was more consistently associated with Fe_IP_ under both water regimes. Flooding–moisture alternation reduced IP formation and lower As availability may have jointly limited As supply and retention on IP (Yu et al., 2024). The stage-dependent responses of As_IP_ to FA, HA, and PT suggest that amendment effects on As_IP_ were associated with changes in As availability and loading behavior, rather than with Fe_IP_ accumulation alone (**Figures 1**, **2**). Continuous flooding may increase As availability and enhance As supply near the rhizosphere, while changes in Fe-bearing retention phases may further alter As loading on IP (Honma et al., 2016; Yuan et al., 2026). The variation in Cd_IP_/Fe_IP_ and As_IP_/Fe_IP_ showed that Cd/As loading relative to plaque Fe did not necessarily follow Fe_IP_ accumulation (**Supplementary Figure S4**). Therefore, organic amendments showed water regime-dependent effects on IP formation and Cd/As retention. Under FM, As_IP_ was positively associated with Fe_IP_, whereas Cd_IP_ showed no significant relationship with Fe_IP_. Under F, both Cd_IP_ and As_IP_ were positively associated with Fe_IP_. Overall, amendment effects on IP-associated Cd/As varied with water regime and growth stage.

### 4.3 Water regime-dependent effects of organic amendments on grain Cd/As accumulation

The metal(loid) accumulation in rice is controlled by both external element supply at the soil–root interface and internal transport processes within rice plants (Li et al., 2022; Zheng et al., 2024). Both water regime and organic amendments may jointly alter Cd/As transport in soil–rice systems, but their effects on Cd/As are usually opposite (Tan et al., 2023; Rumi et al., 2025). In this study, the water regime and organic amendments significantly affected grain Cd/As concentrations and their translocation factors (**Figure 3**; **Supplementary Figure S5**). The distinct responses of grain Cd/As accumulation to the different amendments under the two water regimes were associated with changes in Cd/As availability, IP-mediated Cd/As retention, and translocation within rice tissues, although the relative importance of these processes differed between Cd and As and between water regimes. The RDA further showed corresponding associations among Cd/As availability, metals in IP, and TF_shoot-grain_ of Cd/As (**Figure 7**).

The grain Cd showed weak relationships with CaCl_2_-Cd and Cd_IP_ under FM, but was significantly associated with root Cd and TF_shoot-grain_ of Cd (**Figures 5**, **6**). Moreover, CaCl_2_-Cd was not significantly related to root Cd under either water regime (**Figure 6**), indicating that soil available Cd alone was a poor predictor of root Cd uptake in the present experiment. Previous studies showed that available Cd represents a labile Cd pool in soil, but its relationship with plant Cd accumulation may be modified by rhizosphere pH, root activity, and IP-mediated retention, and internal translocation (Li et al., 2025; Chen et al., 2026). The Cd retained in IP can have dual effects on Cd uptake by rice as a barrier for Cd absorption and a dynamic Cd pool, and such effects may be further amplified by redox fluctuations and organic matter inputs (Liu et al., 2021; Wu et al., 2025). In this study, amendment-induced changes in grain Cd were associated with changes in Cd availability, Cd_IP_, and TF_shoot-grain_ of Cd, but CaCl_2_-Cd alone did not explain root Cd uptake (**Figures 1**, **2**; **Supplementary Figure S5**). Furthermore, the Cd entering rice roots could be transported to aboveground tissues and further redistributed to grains through xylem-to-phloem transfer and remobilization (Zhang et al., 2022b). The organic amendment-derived soluble Cd–organic ligand complexes may further affect Cd uptake and subsequently affect Cd partitioning and translocation within rice plants (Li et al., 2022; Jing et al., 2023). Under F, the grain Cd was decreased and was closely related to Cd in other rice tissues and Cd_IP_ (**Figure 5**). Continuous flooding induced persistently reducing conditions that could decrease Cd mobility and Cd loading on IP (Hao et al., 2026), thereby lowering the external Cd supply to rice roots. Meanwhile, the effects of FA, HA, and PT were more likely expressed through their regulation of Cd retention on IP and exchange at the soil–root interface (**Figure 2**). The grain As was closely associated with available As and As_IP_ under FM (**Figure 5**). Previous studies showed that available As represents a mobile As pool in soil and could strongly affect As uptake by rice, while redox change may further regulate such effect by controlling the release of soil-bound As and transformation of As species (Zhang et al., 2023; Yin et al., 2026). In this study, the close relationship between KH_2_PO_4_-As and root As under FM further indicates that available As was more directly connected with As uptake. Although the IP-mediated As retention is also an important process regulating As transfer within the soil–rice system, redox fluctuations may destabilize IP formation and weaken IP-bound As fixation (Zhang et al., 2023), thereby possibly limiting the As uptake by rice. In this study, the effects of FA, HA, and PT should also be interpreted through their regulation of As_IP_ and TF_shoot-grain_ of As (**Figure 2**; **Supplementary Figure S5**). Under F, the grain As was closely associated with root As and TF_shoot-grain_ As (**Figure 5**). The rice roots could retain a large proportion of As through root sequestration, complexation, and compartmentalization, while organic ligands may modify the As speciation and transport processes (Mawia et al., 2021), thereby affecting the transfer of As to aboveground tissues. In this study, the effects of FA, HA, and PT were more likely mediated by their regulation of TF_shoot-grain_ of As (**Supplementary Figure S5**). Therefore, organic amendments showed water regime-dependent effects on grain Cd/As through different soil–root–grain transfer pathways. The grain Cd under FM and grain As under F were mainly linked to root uptake and shoot-to-grain transfer, whereas grain Cd under F and grain As under FM were mainly related to Cd/As mobility and IP-mediated Cd/As retention.

### 4.4 Implications for grain Cd/As co-reduction and risk management

The contrasting responses of grain Cd/As to organic amendments under the two water regimes highlight the difficulty of achieving simultaneous Cd/As risk reduction in co-contaminated paddy soils (**Figure 3**). Importantly, reductions in grain Cd did not always coincide with reductions in grain As. Under FM, FA and HA increased grain total As by 53.66% and 25.63%, respectively, whereas PT simultaneously reduced grain Cd and As. Under F, PT also increased grain total As by 20.39% despite substantially reducing grain Cd. These results indicate a clear Cd/As trade-off for some amendment–water regime combinations and show that their effects on grain Cd/As concentrations cannot be evaluated on the basis of Cd reduction alone. It is widely recognized that Cd and As usually show distinct responses to redox conditions: redox fluctuations may limit As release but increase Cd availability, whereas reducing conditions tend to decrease Cd mobility while enhancing As mobilization (Li et al., 2026; Yin et al., 2026). This study further showed that grain Cd/As risk was associated not only with soil availability, but also with IP-associated Cd/As retention and TF_shoot-grain_ of Cd/As (**Figure 7**). Thus, reducing Cd/As availability alone may not necessarily ensure lower grain Cd/As accumulation if it simultaneously increases rice uptake or internal translocation.

Water regime and organic amendments were associated with contrasting changes in Cd/As availability and mobility. Continuous flooding induced the persistently reducing conditions that may immobilize Cd while enhancing As release and mobility (Zhang et al., 2022a; Zhang et al., 2023). Furthermore, the organic amendments under such reducing environments may further regulate Cd/As mobility by altering dissolved organic ligands, Fe-associated cycling, and related retention-release reactions (Qiao et al., 2019; Liu et al., 2021; Jin et al., 2023). In contrast, flooding–moisture alternation may limit As release by interrupting reducing conditions, whereas Cd availability may become more sensitive to amendment-induced changes in soil pH, soluble organic ligands, and Cd complexation (Yin et al., 2026; Chen et al., 2026). Collectively, water regime was associated with the general direction of Cd/As mobility, while organic amendments modified the magnitude of these changes.

The IP-mediated Cd/As retention may represent a dynamic soil–root interface process rather than a simple barrier. Although IP has been widely considered an important interface regulating Cd/As transfer from soil to rice roots, its retention function may vary with water regime and organic amendments (Liu et al., 2021; Wu et al., 2025). In this study, the different relationships of Cd_IP_/As_IP_ with Fe_IP_ indicate that IP formation may not be regarded as a uniform barrier for Cd or As. The effectiveness of IP-mediated Cd/As retention depends on the coupled responses of IP formation and Cd/As loading on IP (**Figure 2**), rather than on IP formation alone. Importantly, the effects of the amendments on IP-associated Cd/As retention cannot be evaluated solely from changes in Fe_IP_, but should also consider whether IP-associated Cd/As remains exchangeable and accessible for subsequent uptake by rice roots.

After Cd/As enters rice roots, its internal translocation may become a critical process affecting the grain Cd/As accumulation. Previous studies showed that Cd/As in roots could be redistributed to other tissues and finally accumulate in grains through multiple transfer processes during rice growth (Zhang et al., 2022b). In this study, the close relationships between grain Cd/As and TF_shoot-grain_ of Cd/As indicate that amendment effects on grain Cd/As risk cannot be assessed only by soil immobilization or IP-mediated retention. The organic amendments may reduce Cd/As availability or alter their retention on IP, but they may also influence Cd/As redistribution within rice tissues (Liu et al., 2024). Accordingly, amendment selection should focus not only on reducing soil Cd/As mobility, but also on limiting rice uptake and internal translocation within rice.

The different responses highlight the need for an integrated risk-assessment framework for Cd/As co-contaminated paddy soils. Integrated indices such as SCI and GRI could further link amendment responses with grain Cd/As risk, helping to distinguish simultaneous Cd/As reduction from trade-off responses between the two elements (**Figure 7**). Because inorganic As was not determined, total grain As was conservatively used as a surrogate for inorganic As in the screening-level GRI. Accordingly, the GRI was used for relative comparison among treatments and should not be interpreted as evidence of regulatory exceedance or as an absolute food-safety risk estimate. Based on this framework, the water–amendment combinations were compared according to their effects on the dominant component of grain Cd/As risk rather than on soil availability alone. For Cd-dominant soils, continuous flooding and Cd-targeted amendments have been associated with lower grain Cd concentrations (Tan et al., 2023; Hao et al., 2026), although potential increases in grain As should also be considered. For As-dominant soils, flooding–moisture alternation may limit As mobilization, but its effects on grain Cd concentrations also require consideration (Yin et al., 2026). For Cd/As co-contaminated soils, potential strategies may be evaluated using an integrated risk-assessment framework (SCI, GRI, and Cd/As availability, Cd_IP_/As_IP_, and translocation factors). Overall, the water regime- and amendment-specific concentration responses observed here provide a basis for identifying combinations for further evaluation but do not establish their agronomic suitability. Validation under field conditions, including biomass and grain yield measurements, is therefore required.

As this study was conducted under controlled greenhouse pot conditions during a single rice-growing season, with three biological replicates per treatment and only one soil and one rice cultivar, differences in rhizosphere conditions between pots and field paddies may affect Cd/As behavior and IP formation. Because rice biomass and grain yield were not quantified, the agronomic performance of the amendments remains to be evaluated under field conditions.

## 5 Conclusion

The pot experiment was conducted under two water regimes to evaluate the effects of three organic amendments on Cd/As behavior in the soil–rice system. Results showed that the effects of organic amendments on soil Cd/As availability, IP-mediated Cd/As retention, and associated risk of grain Cd/As accumulation were strongly dependent on water regime. HA reduced soil available Cd under both FM and F. FA reduced available Cd only under F but showed unstable effects under FM. PT only reduced available Cd at the tillering stage and tended to increase available Cd at later stages, whereas available As changed little under FM but increased under F, especially with FA and PT. Water regime also altered Fe_IP_, Cd_IP_, and As_IP_ relationships. Under FM, As_IP_ was positively associated with Fe_IP_, whereas Cd_IP_ was not; under F, both Cd_IP_ and As_IP_ were positively associated with Fe_IP_. The variation in Cd_IP_/Fe_IP_ and As_IP_/Fe_IP_ further showed that Cd/As loading relative to plaque Fe did not necessarily follow Fe_IP_ accumulation. The changes in soil availability and IP-associated retention did not directly translate into grain Cd/As accumulation. PT produced the largest simultaneous decreasesin grain Cd and As concentrations under FM. All amendments reduced grain Cd concentrations under F, while FA reduced grain As concentrations. Grain Cd under FM and grain As under F were mainly linked to root uptake and shoot-to-grain transfer, whereas grain Cd under F and grain As under FM were more related to soil mobility and IP-associated retention. Under the present experimental conditions, the observed amendment effects were limited to grain Cd/As concentrations and screening-level risk indices. Because biomass and grain yield were not measured, agronomic suitability cannot be inferred and requires evaluation under field conditions across different soils and rice cultivars.

## Data Availability

The raw data supporting the conclusions of this article will be made available by the authors, without undue reservation.
