# Supplementary material for: Water regime-dependent effects of organic amendments on Cd/As accumulation and potential grain risk in rice
Source: Front Plant Sci. 2026 Sep 17;17:1942050. doi: 10.3389/fpls.2026.1942050 (PMC13627321; doi:10.3389/fpls.2026.1942050)
Supplement: Supplementary file 1 [file DataSheet1.docx]

**Water regime-dependent effects of organic amendments on Cd/As accumulation and grain risk in rice**

**Authors:** Ping Zhang ^1^, Bo Li ^2*^, Jie Zhang ^2^, Taowen Pan ^2^, Jibiao Geng ^2^, Bingbing Song ^3^, Jianrong Zhao ^1^_,_ Hongbao Wu ^1*^

**Affiliations:**

1 College of Resources and Environment, Anhui Science and Technology University, Chuzhou 233100, China

2 College of Resources and Environment, Linyi University, Key Laboratory of Green Functional Fertilizer Innovation, Ministry of Agriculture and Rural Affairs, Linyi 276000, China

3 Hunan Provincial Ecological Environment Monitoring Center, Changsha 410014, China

***Corresponding author:**

Bo Li

College of Resources and Environment, Linyi University, Key Laboratory of Green Functional Fertilizer Innovation, Ministry of Agriculture and Rural Affairs, Linyi 276000, China

E-mail: libo34@lyu.edu.cn	Fax: +86- 539-7258178	Phone: +86- 539-7258170

Hongbao Wu

College of Resource and Environment, Anhui Science and Technology University, Chuzhou 233100, China. No. 9, Donghua Road, Chuzhou 233100, China

E-mail: wuhb1120@163.com	Fax: +86- 0550-6732656	Phone: +86- 0550-6732036

Supplementary material

**Method S1. Determination methods of soil pH, Eh, total Cd / As, and available Cd / As**

The soil redox potential (Eh) was initially measured at 10 cm below the soil surface using a redox potential depolarization automatic analyzer (FJA, China). Soil pH was measured in a soil-water ratio of 1:2.5 (wt/vol) using a pH meter (PHS-3C, China). Total Cd and As concentrations in soil and organic amendments were digested by HCl-HNO3 (4:1, v/v) with an open system. The soil available Cd and As were extracted by the 0.01 mol L-1 CaCl2 (1:5, w/v) and 0.5 mol L-1 KH_2_PO_4_ (1:15 w / v), respectively, with 120 min shaking. The Cd and As concentrations in all the above digested and extracted supernatants were determined by inductively coupled plasma optical emission spectrometry (ICP-OES; Agilent, USA) and atomic fluorescence spectrophotometry (AFS; AFS-8530, China). Quality control using reference soil GBW07411 (National Standard Soil) yielded Cd and As recoveries of 89.3%–110.2% and 91.1%–105.7%.

**Method S2. Determination methods of iron plaque and Cd / As in rice tissues**

The iron plaque was extracted using the dithionite-citrate-bicarbonate (DCB) method. Approximately 2.0 g of fresh roots rinsed with deionized water was immersed in 40 mL of citrate-bicarbonate extracting solution for 10 min, followed by the addition of 1.0 g sodium dithionite and extraction for another 50 min. The extract was transferred to a 100-mL volumetric flask and diluted to volume with ultrapure water. Fe and Mn in the DCB extracts were determined by ICP-OES (Agilent, USA), whereas Cd and As were determined by ICP-MS (Agilent, USA). Plaque-associated Fe, Mn, Cd, and As were normalized to the dry weight of the corresponding roots.

Rice tissues were digested by microwave-assisted digestion using HNO_3_-H_2_O_2_ (8:1, v/v). After digestion, the solutions were diluted to 10 mL with 1% HNO₃ prior to analysis by ICP-MS (Agilent, USA). Analytical quality control was performed using the certified reference material GBW-07602 (bush twigs and leaves). The recoveries of Cd and As ranged from 96.4% to 105.3% and from 92.1% to 99.5%, respectively.

**Method S3. Determination methods of TOC, TN, TP, and TK**

Total organic carbon (TOC) was determined using K_2_Cr_2_O_7_ oxidation-FeSO_4_ titration method. Total nitrogen (TN) was measured using the Kjeldahl digestion method. Total phosphorus (TP) and total potassium (TK) were determined after H_2_SO_4_-H_2_O_2_ digestion; TP was quantified using the molybdenum-antimony colorimetric method, and TK was quantified by flame photometry.

Fig. S1. Difference analysis of soil pH, Eh, and available Cd/As under different water regimes and organic amendments during key rice growth stages. (a, b) Soil pH under FM and F conditions, respectively; (c, d) soil Eh under FM and F conditions, respectively; (e, f) available Cd under FM and F conditions, respectively; (g, h) available As under FM and F conditions, respectively. Data were compared using a one-way analysis of variance (ANOVA) followed by the least significant difference (LSD) at a probability level of 0.05. Different letters indicate significant differences at *p* < 0.05.

Fig. S2. Soil Cd/As availability reduction rates under different water regimes and organic amendments. (a, b) reduction rates of available Cd under FM and F, respectively; (c, d) reduction rates of available As under FM and F, respectively. FM, flooding-moisture alternation; F, continuous flooding. Error bars represent standard deviations.

Fig. S3. Difference analysis of Fe, Cd, and As in iron plaque (Fe_IP_, Cd_IP_, and As_IP_) under different water regimes and organic amendments during key rice growth stages. (a, b) Fe_IP_ under FM and F conditions, respectively; (c, d) Cd_IP_ under FM and F conditions, respectively; (e, f) As_IP_ under FM and F conditions, respectively. Data were compared using a one-way analysis of variance (ANOVA) followed by the least significant difference (LSD) at a probability level of 0.05. Different letters indicate significant differences at *p* < 0.05.

Fig. S4. Dynamics and multiple comparisons of Cd/Fe and As/Fe ratios in root iron plaque under different water regimes and organic amendments.

Fig. S5. Translocation factors of Cd and As in rice tissues under different water regimes and organic amendments at maturity. (a, b) Cd translocation factors under FM and F, respectively; (c, d) As translocation factors under FM and F, respectively. Different lowercase letters indicate significant differences among amendment treatments within the same tissue and water regime at p < 0.05. Error bars represent standard deviations.

Fig. S6. Dynamic changes in Cd/As contents in rice tissues during rice growth under different water regimes and organic amendments. (a, c, e) Cd contents in roots, stems, and leaves, respectively; (b, d, f) As contents in roots, stems, and leaves, respectively.

Table S1 Basic physicochemical properties of the test soil and materials.

|  | **pH** | **TOC**  **(g kg^-1^)** | **(mg kg^-1^)** | | **Clay %** | **Silt %** | **Sand %** | **(g kg^-1^)** | | | **C/N** |
| --- | --- | --- | --- | --- | --- | --- | --- | --- | --- | --- | --- |
|  |  |  | **Total Cd** | **Total As** |  |  |  | **TN** | **TP** | **TK** |  |
| Soil | 6.01 | 20.06 | 2.13 | 174.05 | 12.80 | 72.45 | 13.75 | 0.71 | 0.73 | 12.65 | 28.25 |
| FA | 5.43 | 580.00 | 0.03 | 0.02 |  |  |  | 106.19 | 1.52 | 28.14 | 5.46 |
| HA | 10.27 | 404.00 | 0.04 | 0.11 | -- | -- | -- | 5.99 | 0.62 | 1.31 | 67.45 |
| PT | 6.82 | 424.00 | 0.07 | 2.24 | -- | -- | -- | 9.17 | 1.09 | 1.99 | 46.24 |

Note: FA, fulvic acid; HA, the humate extracted from woody peat by sodium hydroxide; PT, woody peat; TOC, total organic carbon; TN, total nitrogen; TP, total phosphorus; TK, total potassium. C/N was calculated as the ratio of TOC to TN on a mass basis.

Table S2. Two-way ANOVA results showing the effects of water regime, organic amendment, and their interaction on mature rice Cd/As accumulation, translocation factors, and grain risk indices.

| **Variable** | **Water (W)** | **Amendment (A)** | **W×A** |
| --- | --- | --- | --- |
| Root Cd | *** | *** | *** |
| Stem Cd | *** | ns | ns |
| Leaf Cd | *** | *** | *** |
| Grain Cd | *** | *** | ** |
| Root As | *** | *** | *** |
| Stem As | *** | *** | *** |
| Leaf As | *** | *** | ns |
| Grain As | *** | *** | *** |
| TF_root-stem_ of Cd | *** | *** | *** |
| TF_stem-leaf_ of Cd | *** | *** | *** |
| TF_shoot-grain_ of Cd | *** | *** | *** |
| TF_root-stem_ of As | *** | *** | *** |
| TF_stem-leaf_ of As | ** | *** | *** |
| TF_shoot-grain_ of As | ns | *** | *** |
| Grain Cd reduction rate | *** | *** | *** |
| Grain As reduction rate | *** | *** | *** |
| SCI | *** | *** | *** |
| GRI | *** | *** | *** |

Note: Cd_IP_, As_IP_, and Fe_IP_ indicate Cd, As, and Fe in iron plaque; SCI, grain Cd/As co-reduction index; GRI, Cd/As grain risk index; *** *p* < 0.001, ** *p* < 0.01, * *p* < 0.05, ns: not significant (*p* ≥ 0.05).

Table S3. Two-way ANOVA results showing the effects of water regime, organic amendment, and their interaction on rice tissue Cd/As concentrations at tillering and filling stages.

| **Rice stage** | **Variable** | **Water (W)** | **Amendment (A)** | **W×A** |
| --- | --- | --- | --- | --- |
| Tillering | Root Cd | *** | *** | *** |
|  | shoot Cd | *** | *** | *** |
|  | Root As | *** | *** | ** |
|  | shoot As | *** | ** | * |
| Filling | Root Cd | *** | ** | * |
|  | Stem Cd | *** | *** | ** |
|  | Leaf Cd | *** | *** | *** |
|  | Root As | *** | *** | *** |
|  | Stem As | *** | *** | ** |
|  | Leaf As | *** | *** | *** |

Note: *** *p* < 0.001, ** *p* < 0.01, * *p* < 0.05, ns: not significant (*p* ≥ 0.05).
